# *FMR1* locus isoforms: potential biomarker candidates in fragile X-associated tremor/ataxia syndrome (FXTAS)

**DOI:** 10.1038/s41598-020-67946-y

**Published:** 2020-07-06

**Authors:** Marwa Zafarullah, Hiu-Tung Tang, Blythe Durbin-Johnson, Emily Fourie, David Hessl, Susan M. Rivera, Flora Tassone

**Affiliations:** 1 Department of Biochemistry and Molecular Medicine, School of Medicine, University of California Davis, Sacramento, 95817 CA USA; 2 Division of Biostatistics, School of Medicine, University of California Davis, Davis, CA USA; 3 Center for Mind and Brain, University of California Davis, Davis, CA USA; 4 Department of Psychology, University of California, Davis, Davis, CA USA; 5 MIND Institute, University of California Davis Medical Center, Sacramento, 95817 CA USA; 6 Department of Psychiatry and Behavioral Sciences, University of California Davis Medical Center, Sacramento, 95817 CA USA

**Keywords:** Genetics, Biomarkers

## Abstract

Fragile X associated tremor/ataxia syndrome (FXTAS) is a late adult-onset neurodegenerative disorder that affects movement and cognition in male and female carriers of a premutation allele of 55–200 CGG repeats in the Fragile X mental retardation (*FMR1)* gene. It is currently unknown if and when an individual carrier of a premutation allele will develop FXTAS, as clinical assessment fails to identify carriers at risk before significant neurological symptoms are evident. The primary objective of this study was to investigate the alternative splicing landscape at the *FMR1* locus in conjunction with brain measures in male individuals with a premutation allele enrolled in a very first longitudinal study, compared to age-matched healthy male controls, with the purpose of identifying biomarkers for early diagnosis, disease prediction and, a progression of FXTAS. Our findings indicate that increased expression of *FMR1* mRNA isoforms, including *Iso4/4b*, *Iso10/10b*, as well as of the *ASFMR1 mRNAs Iso131bp*, are present in premutation carriers as compared to non-carrier healthy controls. More specifically, we observed a higher expression of *Iso4/4b and Iso10/10b,* which encode for truncated proteins*,* only in those premutation carriers who developed symptoms of FXTAS over time as compared to non-carrier healthy controls, suggesting a potential role in the development of the disorder. In addition, we found a significant association of these molecular changes with various measurements of brain morphology, including the middle cerebellar peduncle (MCP), superior cerebellar peduncle (SCP), pons, and midbrain, indicating their potential contribution to the pathogenesis of FXTAS. Interestingly, the high expression levels of *Iso4/4b* observed both at visit 1 and visit 2 and found to be associated with a decrease in mean MCP width only in those individuals who developed FXTAS over time, suggests their role as potential biomarkers for early diagnosis of FXTAS.

## Introduction

The fragile X mental retardation (*FMR1*) gene consists of 17 exons spanning approximately 38 kb of genomic DNA. A trinucleotide repeat expansion, greater than 200 CGG, with consequent methylation of the 5′UTR (untranslated region) of *FMR1* gene, leads to Fragile X Syndrome (FXS), the most common form of intellectual disability and known monogenic cause of Autism Spectrum Disorder^1^. Expansions between 55 and 200 CGG repeats (known as premutation carriers) confer the risk of developing Fragile X-associated tremor/ataxia syndrome (FXTAS), a late-onset neurodegenerative disorder characterized by intention tremor, gait ataxia, autonomic dysfunction, and Parkinsonism^2^. In addition, females premutation carriers are at risk of developing Fragile X-associated primary ovarian insufficiency (FXPOI) that affects ovary function in women leading to early menopause and irregular elevation of follicle-stimulating hormone (FSH)^3^. The prevalence of the premutation allele among the general population is 1:110–200 females and 1:430 males with an estimated 40–75% of males and 8–16% of females developing FXTAS^4,5^.

At the molecular level, FXTAS is characterized by an increased level of *FMR1* mRNA containing expanded CGG repeats^6^. The proposed molecular mechanisms of FXTAS pathogenesis include the sequestration of CGG binding proteins by the elevated levels of *FMR1* mRNA leading to RNA toxicity, the production of toxic FMRPolyG protein due to RAN translation and the chronic activation of DNA damage response [reviewed in^7^].

Extensive alternative splicing of the *FMR1* gene has been observed^8–16^. Alternative splicing (AS) is a regulated process occurring during gene expression that increases protein diversity and represents a powerful evolutionary resource. It regulates the protein localization, enzymatic properties, stability, interaction with ligands and membranes^17^ and is common in the nervous system playing a major role in neurogenesis, brain development^18,19^ and cell survival^20^. It is increasingly recognized that disruption of the splicing process, which is regulated by different splicing factors, can contribute to a number of neurological disorders^19,21,22^ including autism spectrum disorder (ASD)^23^, Parkinson’s disease^24^, dementia^25^, spinal muscular atrophy (SMA)^26^, Prader-Willi syndrome (PWS)^27^, schizophrenia^28^, myotonic dystrophy^29^, amyotrophic lateral sclerosis^30,31^ and Alzheimer’s disease^32^.

**Figure 1:**
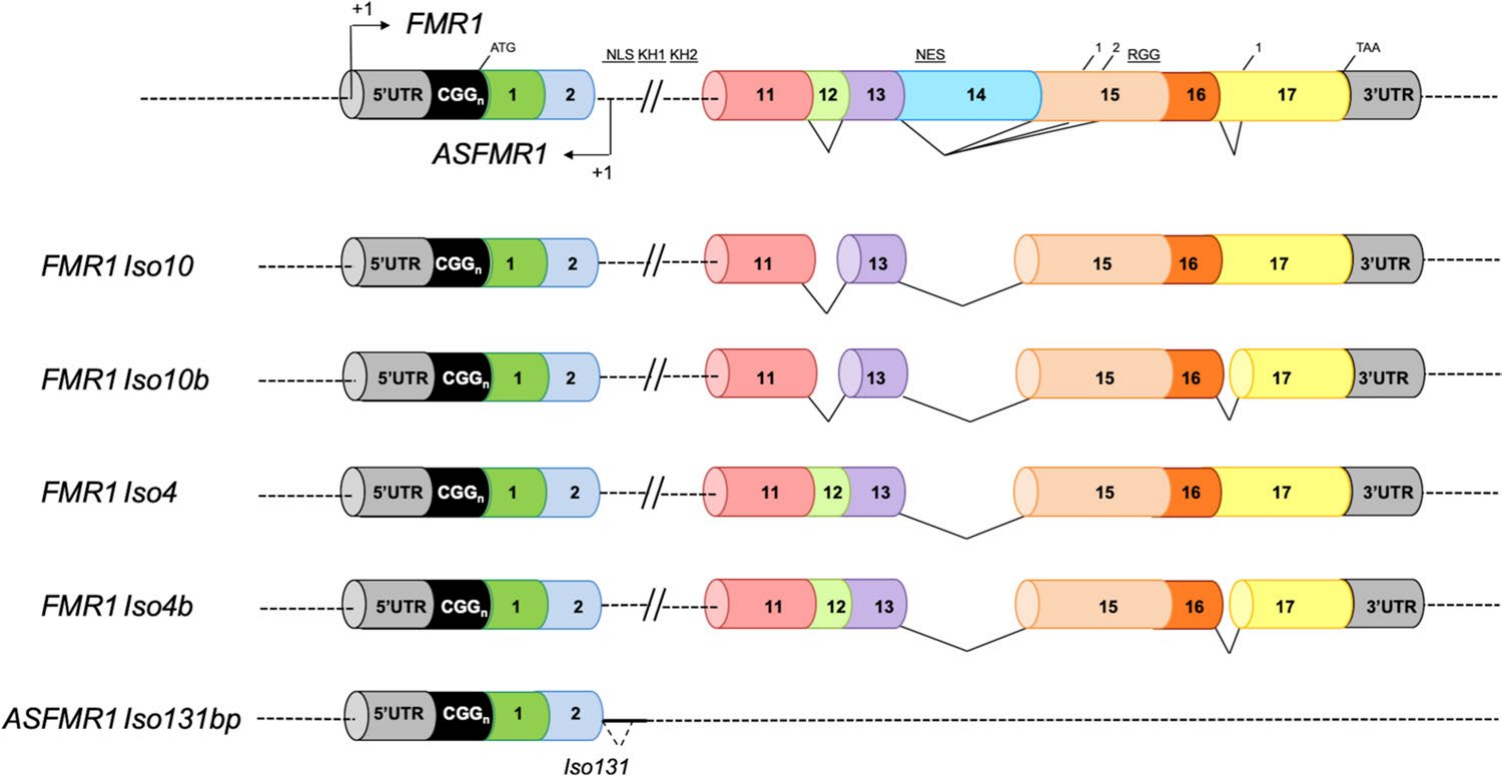
Schematic overview of the *FMR1* and *ASFMR1* isoforms. Diagram representing the *FMR1* locus (top), 4 *FMR1* isoforms (*Iso10, Iso10b, Iso4 and Iso4b)* and the *ASFMR1 Iso131bp*. Exons are represented in different colors and the alternative splice sites are depicted for exon 12, 14,15 and 17. *FMR1* isoforms *Iso10,* and *Iso10b* both miss exon 12 and 14 but differ for the splicing acceptor site in exon 17. Likewise, the *FMR1 Iso4,* and *Iso4b* both miss exon 14 but differ for the splicing acceptor site in exon 17.

In the *FMR1* gene, altered splicing has been observed in premutation carriers where increased levels of the *FMR1* isoforms have been detected^15,16^. Of the many *FMR1* mRNA isoforms that were demonstrated to exist in both humans and mice^11,14–16^, *Iso10/10b* showed the highest levels of expression in premutation samples which, together with the *Iso4/4b* (Fig. 1), result in truncated proteins that lack the function of the nuclear export signal (NES) and RGG box^15^. Of the 49 different *FMR1* isoforms identified, 30 appeared to be expressed only in premutation carriers compared to controls^16^. Additionally, two novel isoforms *IsoPB1.50* and *IsoPB1.21* retain a portion of the intronic sequence between exons 9 and 10, causing a frameshift, which leads to a premature stop codon and consequently encodes for truncated proteins. A differential increase of specific *FMR1* mRNA isoforms has been observed in premutation carriers, suggesting their potential functional relevance in the pathology of FXTAS due to RNA toxicity^15,16^.

Comprehensive analysis of the transcriptional landscape of the human *FMR1* gene revealed the presence of long non-coding RNAs (*FMR4*^33^, *FMR5* and *FMR6*)^34^. Importantly, a unique antisense transcript at the *FMR1* locus (*ASFMR1*), that similarly to the *FMR1* gene, is upregulated in premutations and not expressed in full mutations, has been identified. Thus, the bidirectional expression of the *FMR1* and the *ASFMR1* genes has been suggested to potentially contribute to the clinical phenotype of FXTAS^35^. Interestingly, the *ASFMR1* also exhibits a premutation specific alternative splicing, the *Iso131bp* (Fig. 1)*,* which is mainly expressed in premutation carriers compared to controls, providing a molecular abnormality potentially associated with FXTAS^15,36–38^. However, no studies have been conducted to determine whether altered expression *FMR1* and *ASFMR1 isoforms* are biomarkers of incipient FXTAS, particularly in relation to neurological and neuroanatomical changes.

In this study, we evaluated male premutation carriers enrolled in a longitudinal study at the UC Davis MIND Institute who have been followed for at least two longitudinal time points, and for whom neuroimaging, neuropsychological, and molecular measurements, as well as medical and neurological examinations, were collected. We have recently reported that the middle cerebellar peduncle (MCP) width decreased in a subgroup of these individuals who developed symptoms of FXTAS at subsequent visits (converters) compared to those who did not (non-converters) and compared to normal age-matched controls. Further, we reported reduced midbrain and pons cross-sectional areas in patients with FXTAS compared to both premutation carriers without FXTAS and controls^39^. These regions play an important role within the cortico-cerebellar pathway, which is necessary for the learning and coordination of various movements^40^. Measurements of these areas have been shown previously to successfully differentiate subcortical movement disorders, such as Parkinson’s disease^41^, which presents with tremor similar to that seen in FXTAS.

In the current study, we sought to determine whether the expression levels of alternative splicing isoforms at the *FMR1* locus were significantly different both in premutation carriers who did and did not develop symptoms of FXTAS over time compared to non-carrier healthy controls. In addition, we also investigated whether the changes in molecular measures were associated with changes in brain measures.

## Materials and methods

### Study participants

As part of two continuing longitudinal studies, male participants over the age of 40 years were recruited from the Sacramento, CA area, and throughout the United States and Canada. The study and all experimental protocols were carried out in accordance with the Institutional Review Board (IRB) at the University of California, Davis with written informed consent obtained from all participants in accordance with the Declaration of Helsinki. Participants were fluent in English, with no history of any serious medical or neurological conditions, including a history of alcoholism or drug abuse. FXTAS stage scoring was based on the clinical descriptions as previously described^42^.

Three groups were included in this study: converters, non-converters, and healthy controls. They were matched by age and the length of the interval between visit 1 (V1) and visit 2 (V2); the converters and non-converters groups were also matched by CGG repeat number and they were selected on the basis of the brain measures availability. After two brain scans, on the basis of neurological assessment, FXTAS stage, and CGG repeat length, 15 participants were classified as “*Converters”* as they developed clear FXTAS symptomology between visits (FXTAS stage score was 0–1 at visit 1 and ≥ 2 at visit 2; 15 were defined as “*Non-converters”* because they continued to show no signs of FXTAS at V2 (FXTAS stage score was 0–1 at both V1 and V2) and 15 non-carrier healthy controls (FXTAS stage score was 0 at both V1and V2).

### CGG repeat length

Genomic DNA (gDNA) was isolated from 5 mL of peripheral blood leukocytes using the Gentra Puregene Blood Kit (Qiagen, Valencia, CA, United States). CGG repeat allele size and methylation status were assessed using a combination of Southern Blot analysis and PCR amplification. For Southern blot analysis, 5–10 μg of isolated genomic DNA was digested with EcoRI and NruI, run on an agarose gel, transferred on a nylon membrane and hybridized with the *FMR1*-specific dig-labeled StB12.3 as detailed in^43^. PCR analysis was performed using *FMR1* specific primers (AmplideX PCR/CE, Asuragen, Inc.); amplicons were visualized by capillary electrophoresis, as previously reported an analyzed using Gene Mapper software^44^.

### mRNA expression levels

Total RNA was isolated from 2.5 ml of peripheral blood collected in PAXgene Blood RNA tubes using the PAXgene Blood RNA Kit (Qiagen, Valencia, CA, United States) and quantified using Agilent 2,100 Bioanalyzer system. RNA isolation was performed in a clean and RNA designated area. cDNA was synthesized as previously described^45^. *FMR1* transcript levels measured by performing reverse transcription followed by real-time PCRs (qRT-PCR). qRT-PCR was performed using both Assays-On-Demand from Applied Biosystems (Applied Biosystems, Foster City, CA, United States) and custom-designed TaqMan primers and probe assays,^45^. Probe and primer assays designed to quantify *FMR1* transcripts for the isoform *IsoPB1.21 and IsoPB1.50*, isoform *Iso4/4b* and isoform *Iso10/10b* were as previously reported^16^. Custom designed primers and probe were also designed to quantify the *ASFMR1* gene and *ASFMR1 131 bp* splice isoform^35^.

### Brain measures

The following methods including MRI acquisition and MRPI analysis were originally described in our previous report^39^. High resolution structural magnetic resonance imaging (MRIs) acquisition was obtained on a 3 T Siemens Trio scanner using a 32-channel head coil and a T1-weighted 3D MPRAGE sequence with the following parameters: TR = 2170 ms, TE = 4.86 ms, flip angle = 7º, FoV = 256mm^2^, 192 slices, 1 mm slice thickness. The scans were first aligned along the anterior–posterior commissure line using acpcdetect (https://www.nitrc.org/projects/art)^46^ or manually using DTI Studio (www.mristudio.org)^47^. Then MRI bias field correction was performed using N4 (https://stnava.github.io/ANTs/)^48^.

A series of independent raters (two per measure) who were blinded to the participant age, group, and time point, quantitatively assessed all MR images for four measurements of brain morphology: MCP and superior cerebellar peduncle (SCP) widths as well as pons and midbrain cross-sectional areas based on methods previously described^49,50^ and detailed below.

The pons and midbrain areas were assessed on the mid-sagittal slice, where horizontal lines were drawn through the superior and inferior pontine notches. The midbrain was measured as the area above the superior pontine line – midbrain tegmentum, while the pons was the area between the two horizontal lines of the superior and inferior notches. The width of both left and right MCPs were measured on parasagittal slices. The linear distance of the MCP was delineated by the peripeduncular cerebrospinal fluid spaces of pontocerebellar cisterns, where the pons was still ‘intact’ and the cerebellum was fully formed (white matter connecting the cerebellar tonsil was present). Finally, the widths of both the left and right SCPs were measured on oblique coronal slices, at the midpoint of the SCP, when it first became separated from the inferior colliculi. The linear distance between the medial and lateral SCP borders was measured. For both MCP and SCP widths, a mean score was calculated by averaging the left and right measurements for each participant. The interrater reliability coefficients were excellent, greater than 0.98, for each of the four measurements. The mean score of the raters was used for further analysis.

### Statistical analysis

Statistical Analyses were conducted blind to treatment groups using R, version 3.6.0. Age and CGG repeat numbers were compared between groups using ANOVA F-tests, followed by pairwise comparisons in the event of a significant F test. The association between mRNA expression and CGG repeat length in each group, adjusted for age, was analyzed using linear models with CGG repeat length, group, the interaction between CGG repeat length and group, and age as covariates. mRNA expression was compared between groups, adjusting for age, using linear models with group and age as covariates. The change between timepoint 1 and time point 2 in mRNA expression was compared between groups, adjusting for age and visit interval, using linear models with the group, age, and visit interval as covariates. The association between each brain measure and each molecular measure was analyzed at each time point within each group, adjusting for age, using linear models with molecular measure, group, the group–molecular measure interaction, and age as covariates. The association between changes in brain measures and changes in molecular measures, within each group, adjusting for age and visit interval, was analyzed using linear models including change in molecular measure, group, the group–molecular measure change interaction, age, and visit interval as covariates. mRNA and protein expression values were log-transformed prior to analysis in order to more closely satisfy model assumptions; brain measures were analyzed on their original scale. Due to the exploratory nature of this study, no adjustment for multiple testing was conducted^51,52^. The tables of all analyses conducted are reported in the supplemental materials.

## Results

### Demographics

Numbers of participants (N) for each group, age and CGG repeat number are as reported in (Table 1). Participants ages did not differ significantly between the three groups. CGG repeat numbers were significantly lower in healthy controls than in all other groups, as expected (*p* < 0.001 in both comparisons) and were not significantly different between converters and non-converters (*p* = 0.445).

**Table 1:** Participants baseline characteristics by group.

	Healthy control	Converters	Non-converters	All participants	*p* Value (F-test)
**Age**					
N	15	15	15	45	0.936
Mean (SD)	60.1 (6.7)	61 (6.7)	60.7 (6.4)	60.6 (6.4)	
Median (range)	60 (49–69)	61 (52–72)	62 (47–70)	61 (47–72)	
**CGG**					
N	15	15	15	45	< 0.001
Mean (SD)	28.5 (4.1)	90.1 (22.4)	81.9 (22.1)	66.9 (32.9)	
Median (range)	30 (20–32)	84 (60–141)	78 (56–135)	75 (20–141)	

### *FMR1* mRNA isoforms expression analysis

**Figure 2:**
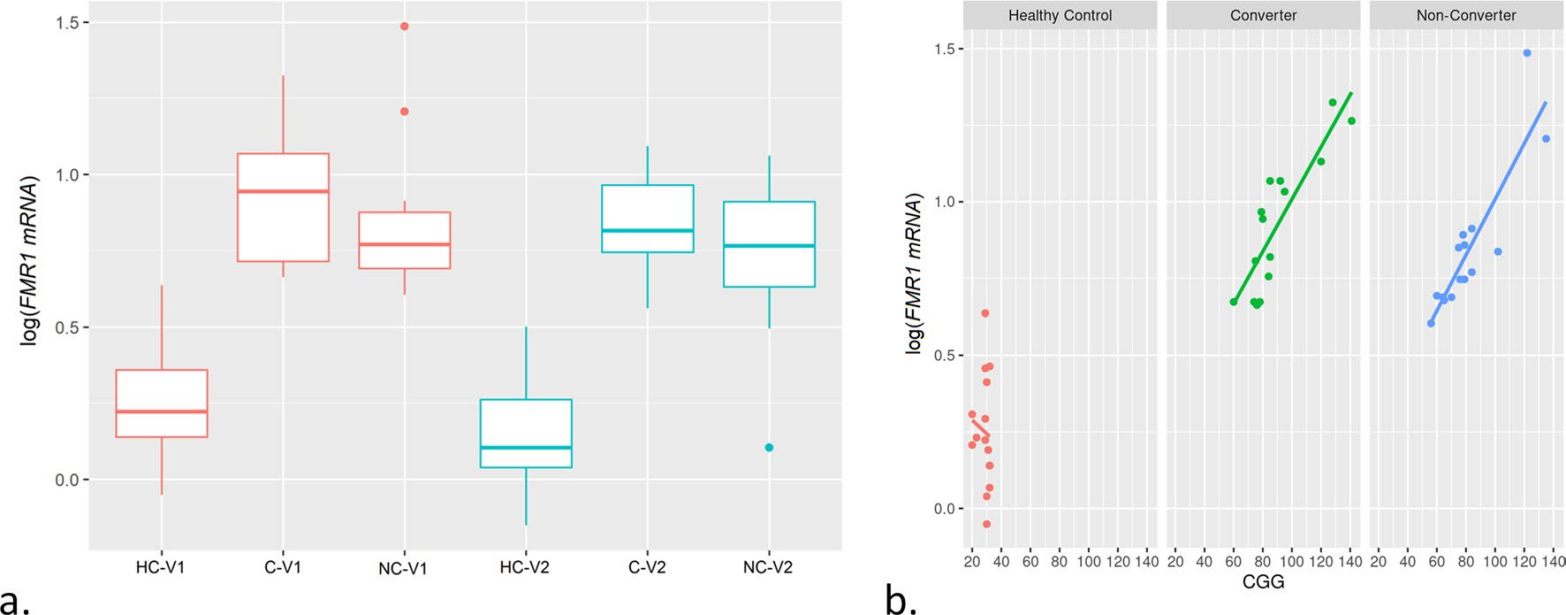
*FMR1* mRNA expression among groups and by CGG repeat length. **a** Box plots showing increased expression levels of *FMR1* mRNA in both premutation groups (converters and non-converters) compared to non-carrier healthy controls at V1 (*p* < 0.001) and V2 (*p* < 0.001) but no significant differences were observed between the two premutation groups. The heavy line in each box represents the median, the lower and upper box edges represent the 25th and 75th percentiles, respectively, and the lower and upper whiskers represent the smallest and largest observations, respectively. **b** Scatter plots showing *FMR1* mRNA expression as function of CGG repeat number at V1 in the three groups.

We measured the expression of *FMR1* mRNA, *FMR1* isoforms *Iso10/10b, Iso4/4b*, *IsoPB1.21, ASFMR1,* and *ASFMR1 Iso131* at V1, V2 and between the visits (Supplementary Material Table S2a, S2b and S2a). As expected, the expression of *FMR1* mRNA was significantly higher in both converters and non-converters as compared to non-carrier healthy controls both at V1 (*p* < 0.001) and V2 (*p* < 0.001) but no significant differences were observed between the two premutation groups (Fig. 2a.). *FMR1* expression levels were associated with CGG repeat length, with greater expression levels being associated with longer CGG repeat length, in both converters and non-converters compared to non-carrier healthy controls (*p* < 0.001 for both comparisons; Fig. 2b.).

**Figure 3:**
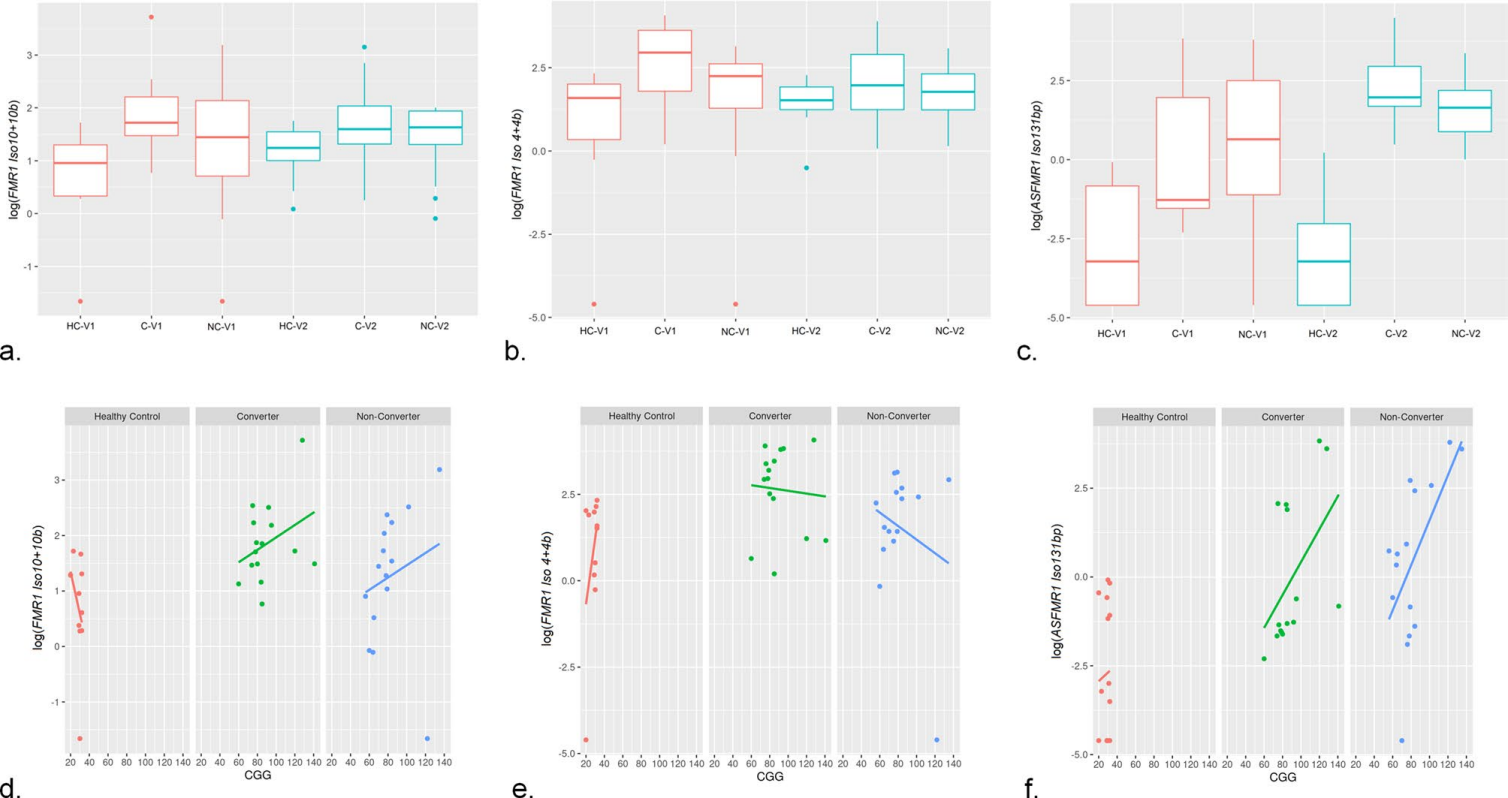
Isoforms mRNA expression levels among three groups and as function of the CGG repeat number. **a **Box plots showing increased levels of *FMR1 Iso10/10b* mRNA at both V1 and V2 (*p* = 0.022 and *p* = 0.046 respectively) only in the converter group but not in the non-converter group (*p* = 0.401 at V1 and *p* = 0.592 at V2) compared to non-carrier healthy controls. **b** Box plots showing increased levels of *FMR1 Iso4/4b mRNA* at V1 (*p* = 0.032) but not at V2 (*p* = 0.247) in the converter group compared to non-carrier healthy controls. No differences were observed in the non-converter group at both visits (*p* = 0.542 at V1 and *p* = 0.684 at V2). **c** Box plots showing increased levels of *ASFMR1 Iso131bp mRNA* at both V1 (*p* < 0.001) and V2 (*p* < 0.001) in both the premutation groups compared to non-carrier healthy controls but not statistically significant differences between the two premutation groups. The heavy line in each box represents the median, the lower and upper box edges represent the 25th and 75th percentiles, respectively, and the lower and upper whiskers represent the smallest and largest observations, respectively. Scatter plots showing **d** *FMR1* **e** of *FMR1 Iso4/4b* and **f** of *ASFMR1 Iso131bp* mRNA expression levels as function of the CGG repeat number at V1 in the three groups.

The expression levels of Iso*10/ Iso10b,* CGG dependent (Fig. 3d) were significantly higher in converters at V1 (*p* = 0.022) and V2 (*p* = 0.046) respectively, as compared to non-carrier healthy controls. Importantly, these markers were not significantly different in non-converters as compared to non-carrier healthy controls at either V1 (*p* = 0.401) or V2 (*p* = 0.592) (Fig. 3a). For *Iso4/4b* we observed significantly higher expression in converters as compared to non-carrier healthy controls at V1 (*p* = 0.032) but no difference was found at V2 (*p* = 0.247) and with the CGG repeat number (Fig. 3e).The expression of these isoforms was not significantly different in non-converters as compared to non-carrier healthy controls both at V1 (*p* = 0.542) and V2 (*p* = 0.684) (Fig. 3b). The expression levels of additional transcripts encoding for truncated proteins, *IsoPB1.21 and IsoPB1.50* were not significantly different among groups both at V1 or V2.

Significantly increased expression levels of *ASFMR1 Iso131* bp, CGG dependent (*p* = 0.012 for the non-converter group and *p* = 0.05 for the converter group; Fig. 3f) were observed in the premutation groups, both converters, and non-converters, as compared to non-carrier healthy controls both at V1 (*p* < 0.001) and V2 (*p* < 0.001) with no difference in levels between the two premutation groups (Fig. 3c).

**Figure 4:**
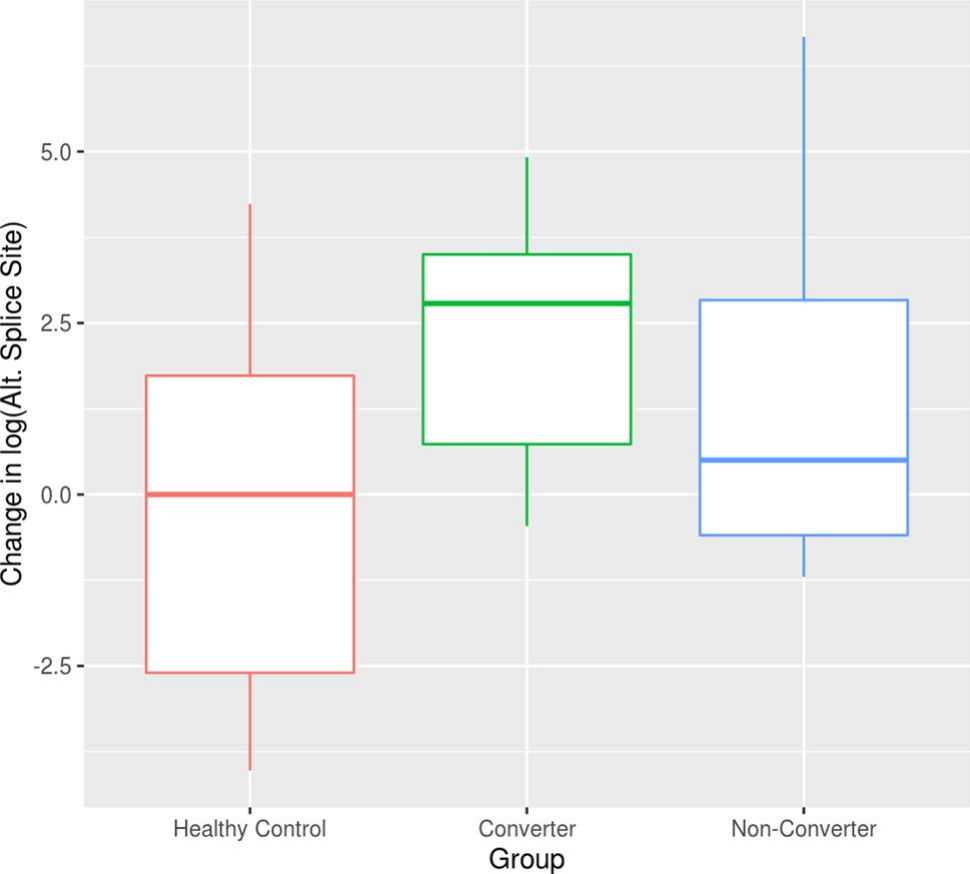
Changes in *ASFMR1 Iso131bp* expression levels between visits. Boxplots showing higher changes between V1 and V2 in the expression level of the *ASFMR1 Iso131bp* in the converter group as compared to non-carrier healthy controls while no significant change in non-converter versus control was observed. The heavy line in each box represents the median, the lower and upper box edges represent the 25th and 75th percentiles, respectively, and the lower and upper whiskers represent the smallest and largest observations, respectively.

However, when comparing the changes in the gene expression levels between V1 and V2 we found a significant greater change in the expression of *ASFMR1 Iso131 bp* in converters (*p* = 0.006; Fig. 4) as compared to non-carrier healthy controls while no significant difference was observed between non-converters and non-carrier healthy controls (*p* = 0.102; Fig. 4).Finally, when comparing the expression levels at V1 and V2 among the groups, no significant differences were detected for any of the *FMR1* splicing isoforms or the *ASFMR1* gene.

### Correlation between brain measures and molecular measures

**Figure 5:**
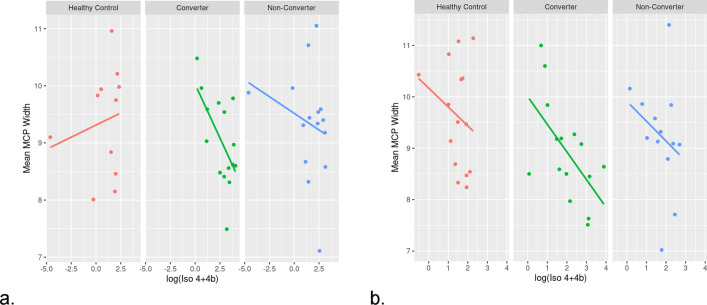
Molecular and brain measure correlations. Scatter plots demonstrating an inverse correlation between the mean MCP width and Iso4/4b in both V1 and V2 but only in the converter group while no significant correlation was observed in the non-converter group.

We compared brain measures, including MCP and SCP width, midbrain and pons cross-sectional area with the molecular measures at V1, V2 and between the visits (Supplementary Material Table S3a, S3B and S3c). Changes in the measures of brain morphology were associated with the expression of some *FMR1* isoforms. Specifically, we found that the higher level of expression of the *Iso4/4b* mRNAs was associated with smaller MCP width in converters both at V1 (*p* = 0.028, beta = -0.421; Fig. 5a). and V2 (*p* = 0.048, beta = -0.531; Fig. 5b) but not in the non-converters at both V1( *p* = 0.611, beta = -0.064; Fig. 5a) or V2 (*p* = 0.333, beta = – 0.392; Fig. 5b) and not in the healthy controls (*p* = 0.530, V1; beta = 0.085; *p* = 0.365, beta = – 0.376). Further, the expression of the *ASFMR1* isoform (131 bp) also increased significantly with a decrease in changes in the pons (*p* = 0.047, beta = – 0.012) only in the converter group between V1 and V2.

Finally, we found a positive correlation, between the change in brain measures, including the mean SCP (*p* = 0.017, beta = 0.726) and midbrain area (*p* = 0.031, beta = 0.316) with increased expression of the *FMR1* mRNA, between the two visits only in the converter group. We also found that the expression of *Iso10/10b* increased significantly with an increase with changes in the midbrain area (*p* = 0.045, beta = 0.076).

## Discussion

Alternative Splicing (AS) is a common process in the central nervous system and crucial for the differentiation and physiology of cells, particularly neurons. It is estimated that AS occurs in about 95% of human genes^53^, contributing greatly to the regulation of mRNA levels and to proteomic diversity. Different studies have reported that AS events take place at the *FMR1* locus and have an impact on the expression and function of FMRP^10–12,14,54–57^ We recently reported on the characterization of the *FMR1* isoforms and showed differential expression and distribution as a function of the CGG repeat number in premutation carriers. Differently from the 24 predicted *FMR1* mRNA variants, we also reported on the existence, of at least 49 different ones in several human tissues, 30 of which detected only in premutation carriers^15,16^. Thus, an altered alternative splicing phenomenon is present in premutation carriers.

In this study, we aimed to identify molecular biomarkers, specifically the expression levels of some alternatively spliced isoforms at the *FMR1* locus, for risk prediction, early diagnosis, and progression of developing FXTAS. Increased and CGG dependent expression levels of *FMR1* mRNA were observed in premutation groups (including both converters and non-converters) as compared to non-carrier healthy controls confirming many previous reports on this well-established altered molecular phenotype in premutation carriers. In addition to *FMR1 mRNA,* we also observed elevated expression of various alternative splicing isoforms as a function of CGG repeat suggesting their potential contribution to the RNA toxicity in premutation carriers.

Taking advantage of the longitudinal study design, we investigated the expression profile for these specific isoforms among our three groups and observed the higher expression of isoform *Iso10/10b* in converters as compared to non-carrier healthy controls at both visits. Importantly, the non-converter group did not show the differential expression for this isoform. This suggests that these isoforms, that encode for truncated proteins might be relevant in the pathogenesis of FXTAS and, pending replication and further confirmation, may eventually play a role in early testing and screening of premutation allele carriers at greater risk of developing the disorder.

Previous investigations on the *FMR1* transcriptional landscape reported the highest expression of isoform *Iso10/10b* in premutation carriers with and without FXTAS^15,16^. However, this study shows a higher expression of these isoforms in individuals, in the converter group, who develop FXTAS over time. Similarly, we observed higher expression of the isoforms *Iso4/4b,* which encode for truncated proteins and are therefore missing the nuclear export signal and C-terminal RGG box, in the converter group, but not in the non-converter group, as compared to healthy controls, also suggesting the potential role of truncated proteins in the pathogenesis of FXTAS, which needs to be further investigated.

The midbrain, pons, and superior cerebellar peduncles (SCP) atrophy is the characteristic pathologic finding in patients with a number of neurodegenerative disorders including progressive supranuclear palsy (PSP)^58,59^, Parkinson's disease (PD), Multiple System Atrophy (MSA)^49,60^ and Alzheimer's disease^61^. These studies have proposed a number of midbrain metrics as potential biomarkers for differentiation of the patients with different neurodegenerative conditions. Shelton et al.^39^ suggested that the decreased MCP width observed in the premutation carriers who developed FXTAS over time (converters) could be a biomarker for early identification of incipient FXTAS. Of relevance, this study found a significant association of increased expression of Iso*4/4b* with decreased MCP width and of the expression of *ASFMR1* isoform (131 bp) with decreased changes in pons, only in the converter group, which supports their potential role as biomarkers and support evidence of their potential contribution to the pathogenesis of the disorder. Interestingly, in the current study we found a positive association of *FMR1* mRNA with mean SCP and midbrain and of isoform *Iso10/10b* with the midbrain, suggesting that some of the molecular measures may be linked with the changes in the brain structures. However, further studies are warranted to confirm these findings.

Generally, the mRNAs containing premature termination codons (PTCs) are degraded by the nonsense-mediated mRNA decay (NMD) system. However, this is not always the case as sometimes the mRNAs escape from the nonsense-mediated mRNA decay and results in truncated proteins^62,63^. Truncated proteins can form aggregates and can act in a dominant-negative manner. The accumulation of these abnormal truncated proteins leads to the gradual loss of function and structure of neurons including the death of neurons. These aggregations have been found to be associated with various neurodegenerative diseases such as Alzheimer’s, Huntington’s and Parkinson’s diseases, ASD, and amyotrophic lateral sclerosis. Additionally, a truncated form of *DISC1* aggregates has been associated with major depression and schizophrenia^64^. Thus, it is possible that the translation of these isoforms might contribute to the pathogenesis of the *FMR1* associated disorders. However, the exact mechanism and functional role are still unknown.

The previous report suggested that the antisense *FMR1* (*ASFMR1*) gene and the premutation specific *ASFMR1 Iso131* bp may contribute to the pathogenesis of FXTAS^35^. Although the expression of this splice isoform in premutation carriers was reported to be higher compared to controls, no difference in the expression levels was observed between non-FXTAS and FXTAS premutation carrier groups in previous studies^36,37^. The study presented here confirms the previous findings of a higher expression of this splice isoform in premutation carriers; however and importantly, the observed significant change of expression levels between V1 and V2 only in the converter group but not in the non-converter or control groups, suggest its potential role in the progression of FXTAS. These findings suggest the presence of an altered bidirectional transcription alternative splicing in premutation carriers and their potential role in the development and progression of FXTAS.

Finally, further studies are required to investigate the function of this specific isoform. The longitudinal follows up of these individuals may help our understanding of the potential role of such isoforms in FXTAS.

## Conclusions

In conclusion, this is a first study that provides evidence of an elevated expression of some of the *FMR1* and *ASFMR1* mRNA splicing isoforms is present in premutation carriers who develop FXTAS compared to those who remain symptom free and to controls, providing support for these measures as potential biomarkers for early identification and monitoring of disease progression. In addition, the association of these molecular measures with brain measures provide us better insight regarding disease pathogenesis. However, due to limitation of the small sample size in the present study, further studies with larger sample size are required to replicate our initial findings and elucidate and confirm the role of these potential biomarkers.

## Supplementary information

Supplementary Data

## Data Availability

All statistical data generated during the study is available in the Additional File.xlsx titled “Supplementary Data”.
